# Safety of inactivated SARS-CoV-2 vaccines in patients with allergic diseases

**DOI:** 10.1186/s12931-022-02054-1

**Published:** 2022-05-27

**Authors:** Chao Cao, Feng Qiu, Chengcheng Lou, Lingling Fang, Fang Liu, Jingjing Zhong, Weijie Sun, Weiping Ding, Xiaopin Yu, Qinhong Xu, Ran Wang, Liemin Ruan, Qifa Song

**Affiliations:** 1 Department of Respiratory and Critical Medicine, Ningbo First Hospital, Ningbo, China; 2 Department of Clinical Laboratory, Ningbo First Hospital, Ningbo, China; 3 Department of Prevention and Healthy Care, Ningbo First Hospital, Ningbo, China; 4 Department of Nursing, Ningbo First Hospital, Ningbo, China; 5 Department of Respiratory and Critical Care Medicine, The First Affiliated Hospital of Anhui Medical University, Hefei, China; 6 Department of Mental Health, Ningbo First Hospital, Ningbo, China; 7 Department of Central Laboratory, Ningbo First Hospital, Ningbo, China

**Keywords:** SARS-CoV-2, Vaccine, Allergic diseases, Safety, Immunogenicity

## Abstract

**Background:**

Considering the considerable prevalence of allergic disease in the general population, an urgent need exists for inactivated SARS-CoV-2 vaccines that can be safely administered to those subjects.

**Methods:**

This retrospective cohort study including 1926 participants who received inactivated SARS-CoV-2 vaccines, compared their local and systemic reactions in 7 days after each dose of inactivated SARS-CoV-2 vaccine, and anti–SARS-CoV-2 IgG after vaccination in all participants.

**Results:**

Pain at the injection site within seven days after the first injection was the most commonly reported local reaction, occurring in 31.0% of the patients with allergic disease and 18.9% in the control group, respectively (P < 0.001). After the first dose, systemic events were more frequently reported in patients with allergic disease than control group (30.2% vs. 22.9%, P < 0.001). After the second dose, systemic events occurred less often, affecting 17.1% of the patients with allergic disease and 11.1% of the control group (P < 0.002). The occurrence of fatigue, vertigo, diarrhea, skin rash, sore throat were the most frequent systemic reactions. Overall, a lower incidence of local and systemic reactive events was observed after the second dose than the first dose in patients with allergic disease and control group. Nearly all participants had positive IgG antibodies, and participants with allergic disease had higher frequencies compared with control group (100.0 vs.99.4%).

**Conclusions:**

Although local and systemic reactions were more frequently reported in patients with allergic disease than control group, administration of the inactivated SARS-CoV-2 vaccine was safe and well tolerated by all participants; no participants experienced a serious adverse event, and none were hospitalized.

*Trial registration:* Chinese Clinical Trial Registry, ChiCTR2100048549. Registered Jul 10, 2021.

**Supplementary Information:**

The online version contains supplementary material available at 10.1186/s12931-022-02054-1.

## Introduction

Severe acute respiratory syndrome coronavirus 2 (SARS-CoV-2) has infected millions of people worldwide[1].Vaccination has proven to be the most effective method for preventing the spread of such an infectious disease. Different formats of SARS-CoV-2 vaccines are currently in development, some of which have been approved by the regulatory authorities and widely used in the community[2–6].Recent reports of reactions to SARS-CoV-2 vaccines have raised questions about their safety for use in individuals with allergies[7].Some patients with allergic disease (AD), such as asthma, allergic rhinitis (AR), allergic dermatitis, had type I hypersensitivity reactions (HR) [8, 9]. These are immune responses that are exaggerated or inappropriate responses to an antigen, which is mediated by IgE antibodies that are produced by the immune system in response to environmental allergens such as pollens, animal danders or dust mites[10–12].These antibodies bind to mast cells and basophils, which contain histamine granules that are released in the reaction and can cause inflammation[13–15].

To date, consistent data are lacking on the need and type of preventive measures in patients with AD receiving SARS-CoV-2 vaccines, especially in those receiving inactivated SARS-CoV-2 vaccines. Considering the extensive prevalence of AD in the general population, an urgent need exists for inactivated SARS-CoV-2 vaccines that can be safely administered to those subjects. Therefore, this study was performed to evaluate the safety of inactivated SARS-CoV-2 vaccines in patients with AD.

## Methods

### Study design, setting and participants

We conducted a retrospective review of the safety among people who work in Ningbo First Hospital after receiving inactivated SARS-CoV-2 vaccines developed by Sinopharm BBIBP-CorV (Sinopharm, Beijing Bio-Institute of Biological Products, containing 6.5U antigen per 0.5 mL) and Sinovac Coronavac (Sinovac Life Sciences, containing 6U antigen per 0.5 mL). The safety was assessed in terms of local or systemic reactogenicity events, which were collected through a series of questionnaires for 7 days after each dose. All participants had negative anti-SARS-CoV-2-serology and nasopharyngeal SARS-CoV-2 real-time RT-PCR swab tests every one to two weeks. Those with a previous SARS-CoV-2 infection, previous coronavirus vaccination, diagnosis of an immunocompromising or immunodeficiency disorder, or receiving immunosuppressant therapy (cytotoxic agents or systemic glucocorticoids) were excluded. The study was approved by the Medical Ethics Commission of Ningbo First Hospital and written informed consent was obtained from each participant. The study was registered with www.chictr.org.cn (ChiCTR2100048549).

All participants answered questions regarding their history of atopic diseases, chronic disease, and long-term medication. The atopic diseases included asthma, allergic rhinitis, allergic dermatitis, eczema, urticaria, etc. The control group (CG) included participants with no history of atopic diseases and no experience of allergies to foods or medicines. Details for recruitment were provided in Additional file 1: Fig. S1. In this survey, the occurrence of local reactions (pain at the injection site, redness, swelling, pruritus, and malaise) and systemic events (fever, fatigue, headache, chills, vomiting, vertigo, somnolence, vomiting, diarrhea, abdominal pain, stuffy nose, chest pain, cardiopalmus, diarrhea, rash, cough, sore throat, chest tightness, new or worsened muscle pain, and new or worsened joint pain) were assessed in the 7 days after receiving each dose of inactivated SARS-CoV-2 vaccine.

Local or systemic adverse reactions were graded according to the FDA Center for Biologics Evaluation and Research (CBER) guidelines on toxicity grading scales and existing, recent studies [2, 3, 16]. The scale of symptoms was self-assessed by the participant and categorized as absent, mild, moderate, severe or worse as following: grade 1 (mild; does not interfere with activity); grade 2 (moderate; interferes with activity), grade 3 (severe; prevents daily activity), and grade 4 (potentially life-threatening; emergency department visit or admission to hospital) (Additional file 1: Tables S1 and S2). Redness and swelling were categorized as grade 1 (2.5-5 cm), grade 2 (5-10 cm), grade 3 (> 10 cm), or grade 4 (necrosis or exfoliation dermatitis) (Additional file 1: Table S1). Fever was defined as an axillary temperature of > 37.0℃. The grades of fever were categorized as 37.1 ~ 38.0℃, 38.1 ~ 39℃, 39.1–40℃ and > 40℃ respectively.

Serum samples were used to measure the concentration of anti-SARS-CoV-2 IgG antibody using the chemiluminescence immunoassay analyzer (Xiamen Innodx Biotech Co., Ltd). According to the manufacturer’s instructions, results with S/CO (sample cut-off value) ≥ 1.0 were regarded as positive[17]. Immunogenicity was defined as post-vaccination positivity of SARS-COV-2 antibody (S/CO ≥ 2) that was weakly positive (1 < S/CO < 2).

## Statistical analysis

The safety analysis included all participants who received both doses of the vaccine. Immunogenicity analysis was based on the participants who completed their assigned two dose vaccination schedule and with available antibody results. Data were analyzed descriptively in an explorative way. The continuous variables were shown as mean ± standard deviation (S.D.), and the categorical variables as numbers and percentages. Pearson χ^2^ test or Fisher’s exact test was used for the analysis of categorical outcomes. The analyses were carried out with R software (version 4.1, R Core Team, 2022; R: A language and environment for statistical computing. R Foundation for Statistical Computing, Vienna, Austria. URL https://www.R-project.org). All tests of hypotheses were considered significant when the two-sided *p*-value was < 0.05.

## Results

Clinical characteristics of the cohort are presented in Table 1. A total of 1926 participants were invited to join this study, among which 381 participants had a history of allergic diseases: allergic rhinitis (n = 236); asthma (n = 15); allergic dermatitis (n = 37); eczema (n = 58); and urticaria (n = 86). Detailed information of allergic status is described in Additional file 1: Table S3. All subjects in this study received two doses of inactivated SARS-CoV-2 vaccine.

**Table 1:** Demographic characteristics of the Participants

Characteristic	Allergic diseases (n = 381)	Control group (n = 1545)
Age, y	35.1 (9.4)	36.7 (11.1)
Sex		
Female	276 (72.4)	969 (62.7)
Male	105 (27.6)	576 (37.3)
Comorbidities		
Hypertension	19 (5.0)	75 (4.9)
Diabetes mellitus	2 (0.5)	13 (0.8)
Chronic gastritis	7 (1.8)	5 (0.3)
Hyperthyroidism	0	5 (0.3)
Hypothyroidism	2 (0.5)	5 (0.3)
Others	2 (0.5)	17 (1.1)
Allergic status		
Asthma	15 (3.9)	
Allergic rhinitis	236 (61.9)	
Atopic dermatitis	37 (9.7)	
Eczema	58 (15.2)	
Urticaria	86 (22.6)	

**Fig. 1:**
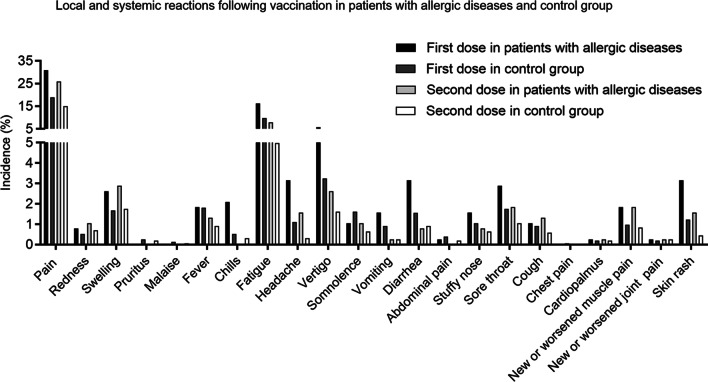
Local and systemic reactions following vaccination in patients with allergic diseases and control group

Administration of the inactivated SARS-CoV-2 vaccine was safe and well tolerated by all the participants, with no participant experiencing a serious event and none was hospitalized. Figure 1 shows the local and systemic reactions occurring within the first week after vaccination. Pain at the injection site within seven days after first injection was the most commonly reported local reaction, occurring in 31.0% of the participants with AD and 18.9% in CG, respectively (*P* < 0.001) (Table 2). After the second injection, 26.0% of the participants with AD reported injection site pain, while 15.0% reported this in the CG group (*P* < 0.001). After the first dose, systemic events were more frequently reported in patients with AD than CG (30.2 vs. 22.9%, *P* < 0.001), including fatigue (16.3 vs. 9.8%, *P* < 0.001), vertigo (5.8 vs. 3.2%, *P* = 0.019), diarrhea (3.1 vs. 1.6%, *P* = 0.039), skin rash (3.2 vs. 1.2%, *P* = 0.008) and sore throat (2.9 vs. 1.7%, *P* = 0.152). After the second dose, systemic events occurred less frequently, affecting 17.1% of the participants with AD and 11.1% of the CG (*P* = 0.002). The occurrence of fatigue (7.9 vs. 5.0%, *P* = 0.027), vertigo (2.6 vs. 1.6%, *P* = 0.188), diarrhea (0.8 vs. 0.9%, *P* = 0.933), skin rash (1.6 vs. 0.5%, *P* = 0.041) and sore throat (1.8 vs. 1.0%, *P* = 0.197) decreased both in participants with AD and CG group after the second injection. Overall, lower rates of local and systemic reactive events were observed after the second dose than that of the first dose in both participants with AD and in the CG.

**Table 2:** Adverse events following vaccination in patients with AD and CG

	After first dose			After second dose		
	AD (n = 381)	CG (n = 1545)	*P* value	AD (n = 381)	CG (n = 1545)	*P* value
No symptoms	181 (47,5)	968 (62.7)	< 0.001	232 (60.9)	1166 (75.5)	< 0.001
Local reaction	124 (32.5)	309 (20.0)	< 0.001	106 (27.8)	253 (16.4)	< 0.001
Pain	118 (31.0)	292 (18.9)	< 0.001	99 (26.0)	232 (15.0)	< 0.001
Redness	3 (0.8)	8 (0.5)	0.806	4 (1.0)	11 (0.7)	0.729
Swelling	10 (2.6)	26 (1.7)	0.224	11 (2.9)	27 (1.7)	0.152
Pruritus	0	4 (0.3)	1.000	0	3 (0.2)	1.000
Malaise	0	2 (0.1)	1.000	0	1 (0.1)	1.000
Systemic reactions	115 (30.2)	354 (22.9)	0.003	65 (17.1)	172 (11.1)	0.002
Fever	7 (1.8)	28 (1.8)	0.974	5 (1.3)	14 (0.9)	0.668
Chills	8 (2.1)	8 (0.5)	0.006	0 (0.0)	5 (0.3)	0.590
Fatigue	62 (16.3)	151 (9.8)	< 0.001	30 (7.9)	77 (5.0)	0.027
Headache	12 (3.1)	17 (1.1)	0.003	6 (1.6)	5 (0.3)	0.012
Vertigo	22 (5.8)	50 (3.2)	0.019	10 (2.6)	25 (1.6)	0.188
Somnolence	4 (1.0)	25 (1.6)	0.561	4 (1.0)	10 (0.6)	0.623
Vomiting	6 (1.6)	14 (0.9)	0.249	1 (0.3)	4 (0.3)	1.000
Diarrhea	12 (3.1)	24 (1.6)	0.039	3 (0.8)	14 (0.9)	0.933
Abdominal pain	1 (0.3)	6 (0.4)	0.913	0	3 (0.2)	1.000
Stuffy nose	6 (1.6)	16 (1.0)	0.375	3 (0.8)	10 (0.6)	0.960
Sore throat	11 (2.9)	27 (1.7)	0.152	7 (1.8)	16 (1.0)	0.197
Cough	4 (1.0)	14 (0.9)	0.971	5 (1.3)	9 (0.6)	0.244
Chest pain	0	1 (0.1)	1.000	0	0	1.000
Cardiopalmus	1 (0.3)	3 (0.2)	0.586	1 (0.3)	3 (0.2)	0.586
New or worsened muscle pain	7 (1.8)	15 (1.0)	0.154	7 (1.8)	13 (0.8)	0.151
New or worsened joint pain	1 (0.3)	3 (0.2)	0.586	1 (0.3)	4 (0.3)	1.000
Skin rash	12 (3.1)	19 (1.2)	0.008	6 (1.6)	7 (0.5)	0.041

**Fig. 2:**
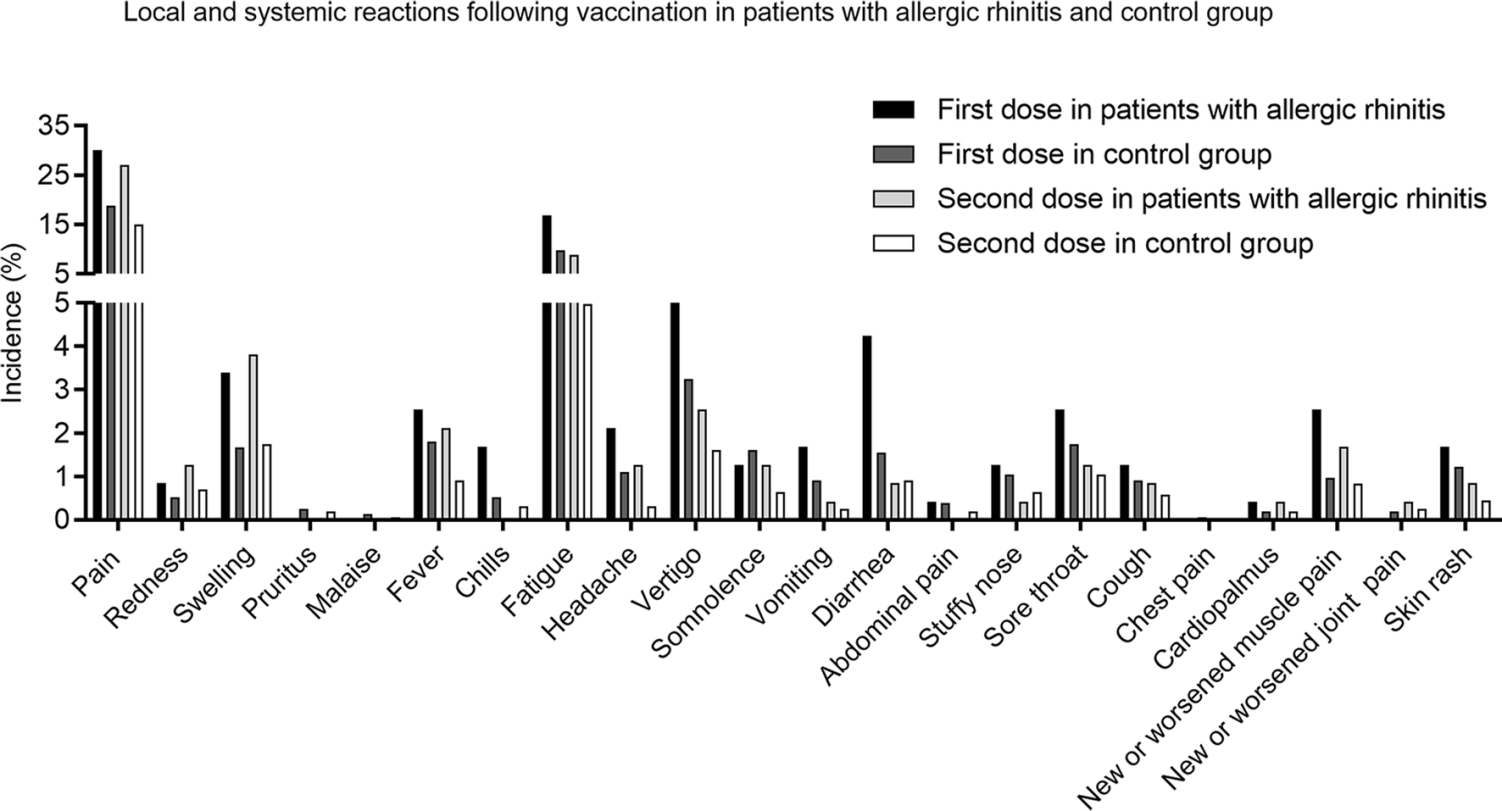
Local and systemic reactions following vaccination in patients with allergic rhinitis and control group

Among participants with AR, the most frequently reported vaccine reactions were pain at the injection site after the first dose (30.1 vs. 18.9%, *P* < 0.001) (Fig. 2; Table 3). After the second dose, pain at the injection site was present in 27.1% of the participants with AR. Systemic events were reported in 30.5 and 17.8% of the participants with AR after the first and second doses, respectively. Fatigue (16.9 vs. 9.8%, *P* < 0.001), vertigo (5.1 vs. 3.2%, *P* = 0.149) and diarrhea (4.2 vs. 1.6%, *P* = 0.011) were the most frequent reactions after the first injection. As predicted, fatigue (8.9 vs. 5.0%, *P* = 0.014), vertigo (2.5 vs. 1.6%, *P* = 0.312) and diarrhea (0.8 vs. 0.9%, *P* = 0.778) occurred less often after the second injection.

**Table 3:** Adervse events following vaccination in patients with AR and CG

	AR (n = 236)	CG (n = 1545)	*P* value	AR (n = 236)	CG (n = 1545)	*P* value
No symptoms	114 (48.3)	968 (62.7)	< 0.001	141 (59.7)	1166 (75.5)	< 0.001
Local reaction	75 (31.8)	309 (20.0)	< 0.001	69 (29.2)	253 (16.4)	< 0.001
Pain	71 (30.1)	292 (18.9)	< 0.001	64 (27.1)	232 (15.0)	< 0.001
Redness	2 (0.8)	8 (0.5)	0.870	3 (1.3)	11 (0.7)	0.610
Swelling	8 (3.4)	26 (1.7)	0.126	9 (3.8)	27 (1.7)	0.064
Pruritus	0	4 (0.3)	1.000	0	3 (0.2)	1.000
Malaise	0	2 (0.1)	1.000	0	1 (0.1)	1.000
Systemic reactions	72 (30.5)	354 (22.9)	0.011	42 (17.8)	172 (11.1)	0.003
Fever	6 (2.5)	28 (1.8)	0.611	5 (2.1)	14 (0.9)	0.177
Chills	4 (1.7)	8 (0.5)	0.103	0	5 (0.3)	1.000
Fatigue	40 (16.9)	151 (9.8)	< 0.001	21 (8.9)	77 (5.0)	0.014
Headache	5 (2.1)	17 (1.1)	0.316	3 (1.3)	5 (0.3)	0.132
Vertigo	12 (5.1)	50 (3.2)	0.149	6 (2.5)	25 (1.6)	0.312
Somnolence	3 (1.3)	25 (1.6)	0.906	3 (1.3)	10 (0.6)	0.523
Vomiting	4 (1.7)	14 (0.9)	0.436	1 (0.4)	4 (0.3)	0.509
Diarrhea	10 (4.2)	24 (1.6)	0.011	2 (0.8)	14 (0.9)	0.778
Abdominal pain	1 (0.4)	6 (0.4)	1.000	0	3 (0.2)	1.000
Stuffy nose	3 (1.3)	16 (1.0)	0.990	1 (0.4)	10 (0.6)	0.970
Sore throat	6 (2.5)	27 (1.7)	0.559	3 (1.3)	16 (1.0)	0.990
Cough	3 (1.3)	14 (0.9)	0.859	2 (0.8)	9 (0.6)	0.970
Chest pain	0	1 (0.1)	1.000	0	0	1.000
Cardiopalmus	1 (0.4)	3 (0.2)	0.434	1 (0.4)	3 (0.2)	0.434
New or worsened muscle pain	6 (2.5)	15 (1.0)	0.079	4 (1.7)	13 (0.8)	0.370
New or worsened joint pain	0	3 (0.2)	1.000	1 (0.4)	4 (0.3)	0.509
Skin rash	4 (1.7)	19 (1.2)	0.780	2 (0.8)	7 (0.5)	0.762

**Fig. 3:**
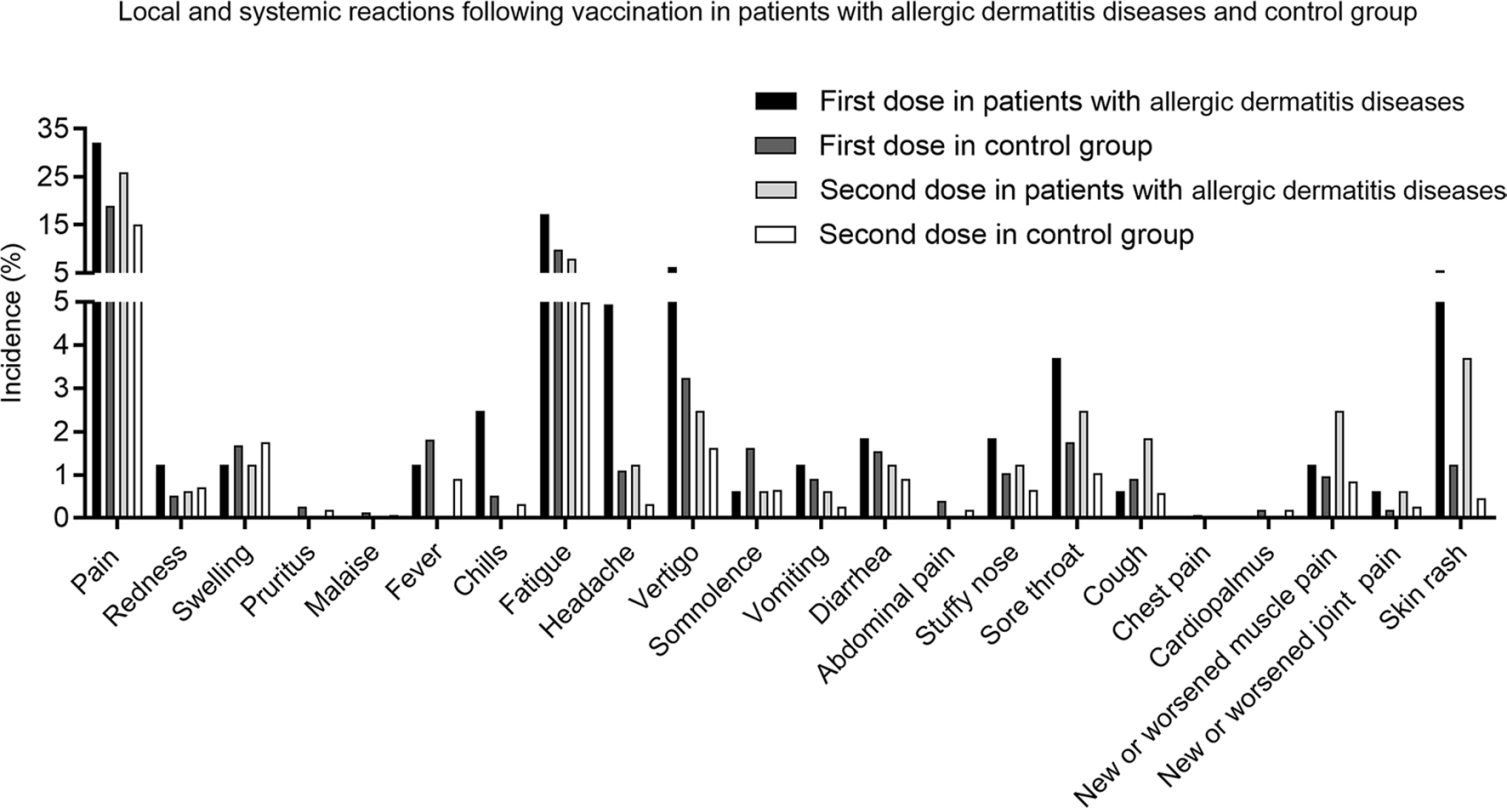
Local and systemic reactions following vaccination in patients with allergic dermatitis diseases and control group

There were 162 participants with ADD (allergic dermatitis disease) enrolled in this study. After the first dose, the most frequently reported vaccine reactions in participants with ADD were pain at the injection site (32.1% *vs.* 18.9%, *P* < 0.001), fatigue (17.3% *vs.* 9.8%, *P* = 0.003), vertigo (6.2% *vs.* 3.2%, *P* = 0.054), headache (4.9% *vs.* 1.1%, *P* < 0.001), skin rash (5.6% *vs.* 1.2%, *P* < 0.001) and sore throat (3.7% *vs.* 1.7%, *P* = 0.155) (Fig. 3; Table 4). Similarly, after the second dose, fatigue (8.0% *vs.* 5.0%, *P* = 0.099), vertigo (2.5% *vs.*1.6%, *P* = 0.633), headache (1.2% *vs.* 0.3%, *P* = 0.137), skin rash (3.7% *vs.* 0.5%, *P* < 0.001) and sore throat (2.5% *vs.* 1.0%, *P* = 0.219) decreased in participants with ADD (Table 4). For participants with eczema and urticaria, fatigue, headache, sore throat, skin rash, and vertigo were the most commonly reported systemic reactions after the first dose (Additional file 1: Table S4). However, no headache or vertigo was observed following the second dose in participants with eczema. For patients with urticaria, new or worsened muscle pains were slightly increased after the second injection, affecting 4.7% of participants.

**Table 4:** Adverse events following vaccination in patients with allergic dermatitis diseases

	After first dose			After second dose		
	ADD (n = 162)	CG (n = 1545)	*P* value	ADD (n = 162)	CG (n = 1545)	*P* value
No symptoms	72 (44.4)	968 (62.7)	< 0.001	96 (59.3)	1166 (75.5)	< 0.001
Local reaction	55 (34.0)	309 (20.0)	< 0.001	44 (27.2)	253 (16.4)	< 0.001
Pain	52 (32.1)	292 (18.9)	< 0.001	42 (25.9)	232 (15.0)	< 0.001
Redness	2 (1.2)	8 (0.5)	0.244	1 (0.6)	11 (0.7)	0.721
Swelling	2 (1.2)	26 (1.7)	0.919	2 (1.2)	27 (1.7)	0.872
Pruritus	0	4 (0.3)	1.000	0	3 (0.2)	1.000
Malaise	0	2 (0.1)	1.000	0	1 (0.1)	1.000
Systemic reactions	51 (31.5)	354 (22.9)	0.015	30 (18.5)	172 (11.1)	0.005
Fever	2 (1.2)	28 (1.8)	0.827	0	14 (0.9)	0.448
Chills	4 (2.5)	8 (0.5)	0.020	0	5 (0.3)	1.000
Fatigue	28 (17.3)	151 (9.8)	0.003	13 (8.0)	77 (5.0)	0.099
Headache	8 (4.9)	17 (1.1)	< 0.001	2 (1.2)	5 (0.3)	0.137
Vertigo	10 (6.2)	50 (3.2)	0.054	4 (2.5)	25 (1.6)	0.633
Somnolence	1 (0.6)	25 (1.6)	0.514	1 (0.6)	10 (0.6)	0.638
Vomiting	2 (1.2)	14 (0.9)	0.987	1 (0.6)	4 (0.3)	0.393
Diarrhea	3 (1.9)	24 (1.6)	0.967	2 (1.2)	14 (0.9)	0.987
Abdominal pain	0	6 (0.4)	1.000	0	3 (0.2)	1.000
Stuffy nose	3 (1.9)	16 (1.0)	0.583	2 (1.2)	10 (0.6)	0.721
Sore throat	6 (3.7)	27 (1.7)	0.155	4 (2.5)	16 (1.0)	0.219
Cough	1 (0.6)	14 (0.9)	0.946	3 (1.9)	9 (0.6)	0.178
Chest pain	0	1 (0.1)	1.000	0	0 (0.0)	1.000
Cardiopalmus	0	3 (0.2)	1.000	0	3 (0.2)	1.000
New or worsened muscle pain	2 (1.2)	15 (1.0)	0.925	4 (2.5)	13 (0.8)	0.117
New or worsened joint pain	1 (0.6)	3 (0.2)	0.329	1 (0.6)	4 (0.3)	0.393
Skin rash	9 (5.6)	19 (1.2)	< 0.001	6 (3.7)	7 (0.5)	< 0.001

The concentration of anti-SARS-CoV-2 IgG antibody in serum samples were measured 4 weeks after the second dose. There were 83 participants with AD and 308 participants in the CG group with available antibody results. Nearly all participants had positive antibodies and participants with AD had higher frequencies (83 (100.0%) versus 306 (99.4%)), and had higher levels of S/CO ≥ 2 (79 (95.2%) versus 277 (89.9%), *P* = 0.192) compared with the CG (Additional file 1: Table S5).

## Discussion

In this large sample size study of inactivated SARS-CoV-2 vaccine in participants with AD, we found a good safety profile with no serious local or systemic reactogenicity events related to the vaccine. The immunogenicity rate was similar in patients with AD when compared with that of the control group. To the best of our knowledge, this is the first study to evaluate the safety and immunogenicity of inactivated SARS-CoV-2 vaccines in patients with AD.

This study enrolled 381 participants with AD and 1,545 healthy individuals. The results showed local and systemic reactions were more frequently reported in participants with AD than controls. Pain at the injection site was the most commonly reported local reaction throughout all participants. Within the control group, local and systemic reactions after the first dose occurred in 20.0% and 22.9% of participants, respectively. In a recent study, the occurrence of local reactions (19.8%) is consistent with our findings in the control group, but higher incidences of systemic reactions (33.5%) were observed in their study[3]. This difference was likely attributable to our study having a control group with fewer comorbidities and age differences in participants; our enrolled control group were younger (50.0 vs. 36.7) [3]. Vaccine safety data in participants with AD were not reported in this previous study.

Systemic events were more frequently reported in participants with AD than the CG. Fatigue, vertigo, diarrhea, skin rash and sore throat were five most frequently occurring systemic reactions in participants with AD after the first dose. The local and systemic reactions of 236 participants with AD and 162 patients with ADD were further analyzed. Among participants with AR, fatigue, vertigo and diarrhea were the most frequent systemic reactions. As expected, skin rash was one of the common systemic reactions in patients with ADD. Besides skin rash, fatigue, vertigo, headache, and sore throat were other major systemic reactions observed in participants with AD. It should be noted that most systemic reactions decreased after the second dose.

The other notable finding in our study was that good antibody responses with inactivated SARS-CoV-2 vaccine were observed in participants with AD. A higher overall rate of positive IgG antibodies was observed in participants with AD (95.2%) when compared to rates of the general population (89.9%). However, this difference did not reach statistical significance; it could be attributed to more immunogenic reactions in this participant group. Another possible explanation is that there is a relationship between local and systemic reactions and antibody response after the complete vaccination course. In the study by Zitt et al.[18], the investigators also found those who reported more local reaction had numerically higher antibody concentrations than those without.

A strength of our research is that all participants were health care workers. Therefore, they could accurately describe whether they had AD or other diseases when completing the questionnaire. Moreover, they can better assess the occurrence and severity of the local and systemic reactions after vaccination than the general population. In addition, to exclude individuals whose immune systems might have been stimulated before and during the study, all enrolled participants received negative results from anti-SARS-CoV-2-serology and nasopharyngeal SARS-CoV-2 real-time RT-PCR swab tests that were taken every one to two weeks throughout the study period.

The current study also has some imitations. First, the data obtained from retrospective questionnaires may have been biased towards capturing more serious reactions and could have missed mild reactions or reactions that did not come to the attention of the participants. Second, age is a well known influential factor of vaccination response[19]; most participants in this study was less than 60 years old due to the characteristics of the study setting. Third, the sample size was not large enough. A larger number of participants may have allowed us to discover the possibility of serious adverse reactions in some uncertain situations.

## Conclusions

In summary, whether inactivated SARS-CoV-2 vaccine is safe in patients with AD has become a substantial clinical concern. Our data indicate an increase in the incidence of local and systemic reactions in participants with AD. Nonetheless, no participants experienced a serious adverse event, and none was hospitalized. Local and systemic reactions were less frequently reported in all participants after the second dose than the first. Based on these findings, we support the current Canadian Society of Allergy and Clinical Immunology (CSACI) recommendations for the SARS-CoV-2 vaccine in individuals with allergies. Administration of the inactivated SARS-CoV-2 vaccine was safe and well tolerated in participants with AD.

## Supplementary Information

**Additional file 1. Table S1.** Local reastion grading scale. **Table S2.** Systemic event grading scale. **Table S3.** Detailed information of allergic status. **Table S4. **Local and systemic reactions following vaccination among patients with eczema, uriticaria, aoptic dermatitis, and asthma. **Table S5.** Anti-SARS-CoV-2 IgG after 4 weeks of the second dose of vaccination in patients with AD and CG. **Figure S1.** The diagram depicts the enrollment and analysis of participants.

## Data Availability

The datasets used and analyzed during the current study are available from the corresponding author on reasonable request.

## Abbreviations

SARS-CoV-2: Severe acute respiratory syndrome coronavirus 2
AD: Allergic disease
AR: Allergic rhinitis
AD: Allergic dermatitis
HR: Hypersensitivity reactions
CG: Control group
CBER: Center for Biologics Evaluation and Research
SD: Standard deviation
CSACI: Canadian Society of Allergy and Clinical Immunology
