# Supplementary material for: Safety of inactivated SARS-CoV-2 vaccines in patients with allergic diseases
Source: Respir Res. 2022 May 27;23:133. doi: 10.1186/s12931-022-02054-1 (PMC9137440; doi:10.1186/s12931-022-02054-1)

| **Additional file 1 Table S1.** Local Reastion Grading Scale | | | |  |
| --- | --- | --- | --- | --- |
|  | **Mild**  **(Grade 1)** | **Moderate (Grade 2)** | **Severe (Grade 3)** | **Potentially Life Threatening (Grade 4)** |
| **Pain** | Does not interefere with activity | Intereferes with activity | Prevents daily activity | Emergency room visit or hospitalization for severe pain |
| **Pruritus** | Does not interefere with activity | Intereferes with activity | Prevents daily activity | Emergency room visit or hospitalization for severe pain |
| **Malaise** | Does not interefere with activity | Intereferes with activity | Prevents daily activity | Emergency room visit or hospitalization for severe pain |
| **Redness** | 2.5 cm ~ 5.0 cm | >5 cm ~ 10.0 cm | >10 cm | Necrosis or exfoliative dermatitis |
| **Swelling** | 2.5 cm ~ 5.0 cm | >5 cm ~10.0 cm | >10 cm | Necrosis or exfoliative dermatitis |

| **Additional file 1 Table S2.** Systemic Event Grading Scale | | | |  |
| --- | --- | --- | --- | --- |
|  | **Mild**  **(Grade 1)** | **Moderate (Grade 2)** | **Severe (Grade 3)** | **Potentially Life Threatening (Grade 4)** |
| **Vomiting** | 1~2 times/24 hours | >2 times/ 24 hours | Requires IV hydration | Emergency room visit or hospitalization for severe pain |
| **Diarrhea** | 2~3 loose stools/24 hours | 4~5 loose stools/24 hours | >6 loose stools/24 hours | Emergency room visit or hospitalization for severe pain |
| **Abdominal pain** | Does not interefere with activity | Some intereferes with activity | Prevents daily activity | Emergency room visit or hospitalization for severe pain |
| **Headache** | Does not interefere with activity | Some intereferes with activity | Prevents daily activity | Emergency room visit or hospitalization for severe pain |
| **Vertigo** | Does not interefere with activity | Some intereferes with activity | Prevents daily activity | Emergency room visit or hospitalization for severe pain |
| **Somnolence** | Does not interefere with activity | Some intereferes with activity | Prevents daily activity | Emergency room visit or hospitalization for severe pain |
| **Fatigue** | Does not interefere with activity | Some intereferes with activity | Prevents daily activity | Emergency room visit or hospitalization for severe pain |
| **Chills** | Does not interefere with activity | Some intereferes with activity | Prevents daily activity | Emergency room visit or hospitalization for severe pain |
| **Cough** | Does not interefere with activity | Some intereferes with activity | Prevents daily activity | Emergency room visit or hospitalization for severe pain |
| **Sore throat** | Does not interefere with activity | Some intereferes with activity | Prevents daily activity | Emergency room visit or hospitalization for severe pain |
| **Stuffy nose** | Does not interefere with activity | Some intereferes with activity | Prevents daily activity | Emergency room visit or hospitalization for severe pain |
| **Cardiopalmus** | Does not interefere with activity | Some intereferes with activity | Prevents daily activity | Emergency room visit or hospitalization for severe pain |
| **Chest pain** | Does not interefere with activity | Some intereferes with activity | Prevents daily activity | Emergency room visit or hospitalization for severe pain |
| **Skin rash** | <10% BSA | 10~30% BSA | >30% BSA |  |
| **New or worsened muscle pain** | Does not interefere with activity | Some intereferes with activity | Prevents daily activity | Emergency room visit or hospitalization for severe pain |
| **New or worsened joint pain** | Does not interefere with activity | Some intereferes with activity | Prevents daily activity | Emergency room visit or hospitalization for severe pain |

**Additional file 1 Table S3.** Detailed information of allergic status

| **Nubmers** | **Allergic rhinitis** | **Urticaria** | **Eczema** | **Aoptic  dermatitis** | **Asthma** | **Allergic conjunctivitis** |
| --- | --- | --- | --- | --- | --- | --- |
| 206 | + | - | - | - | - | - |
| 66 | - | + | - | - | - | - |
| 37 | - | - | + | - | - | - |
| 21 | - | - | - | + | - | - |
| 8 | - | - | - | - | + | - |
| 1 | - | - | - | + | - | + |
| 6 | + | + | - | - | - | - |
| 7 | + | - | + | - | - | - |
| 5 | + | - | - | + | - | - |
| 4 | + | - | - | - | + | - |
| 1 | + | - | - | - | - | + |
| 2 | + | + | + | - | - | - |
| 1 | + | + | + | + | - | - |
| 1 | + | + | + | - | + | - |
| 1 | + | + | - | + | - | - |
| 1 | + | - | + | + | - | - |
| 1 | + | - | + | - | + | - |
| 2 | - | + | - | + | - | - |
| 2 | - | + | + | + | - | - |
| 3 | - | + | + | - | - | - |
| 1 | - | + | - | - | + | - |
| 1 | - | + | - | - | - | + |
| 3 | - | - | + | + | - | - |
|  |  |  |  |  |  |  |

**Additional file 1 Table S4.** Local and systemic reactions following vaccination among patients with eczema, uriticaria, aoptic dermatitis, and asthma

|  | **After first dose** | | | |  | **After second dose** | | | |
| --- | --- | --- | --- | --- | --- | --- | --- | --- | --- |
|  | **Eczema  (*n*=58)** | **Urticaria  (n=86)** | **Aoptic dermatitis (n=37)** | **Asthma  (n=15)** |  | **Eczema  (n=58)** | **Urticaria  (n=86)** | **Aoptic dermatitis (n=37)** | **Asthma  (n=15)** |
| **No symptoms** | 24(41.4) | 36(41.9) | 16(43.2) | 6(40.0) |  | 28(48.3) | 50(58.1) | 26(70.3) | 8(53.3) |
| **Local reaction** | 18(31.0) | 31(36.0) | 9(24.3) | 5(33.3) |  | 23(39.7) | 21(24.4) | 5(13.5) | 5(33.3) |
| **Pain** | 16(27.6) | 30(34.9) | 9(24.3) | 5(33.3) |  | 21(36.2) | 21(24.4) | 5(13.5) | 5(33.3) |
| **Redness** | 1(1.7) | 1(1.2) | 0 | 0 |  | 0 | 1(1.2) | 0 | 0 |
| **Swelling** | 1(1.7) | 1(1.2) | 0 | 0 |  | 2(3.4) | 0 | 0 | 0 |
| **Systemic reactions** | 20(34.5) | 30(34.9) | 13(35.1) | 6(40.0) |  | 11(19.0) | 21(24.4) | 8(21.6) | 3(20.0) |
| **Fever** | 1(1.7) | 2(2.3) | 0 | 0 |  | 0 | 0 | 0 | 0 |
| **Chills** | 2(3.4) | 3(3.5) | 0 | 1(6.7) |  | 0 | 0 | 0 | 0 |
| **Fatigue** | 14(24.1) | 14(16.3) | 6(16.2) | 4(26.7) |  | 5(8.6) | 8(9.3) | 3(8.1) | 3(20.0) |
| **Headache** | 5(8.6) | 7(8.1) | 1(2.7) | 0(0.0) |  | 0 | 1(1.2) | 1(2.7) | 1(6.7) |
| **Vertigo** | 3(5.2) | 8(9.3) | 1(2.7) | 1(6.7) |  | 0 | 4(4.7) | 1(2.7) | 1(6.7) |
| **Somnolence** | 0 | 0 | 1(2.7) | 0 |  | 1(1.7) | 0 | 0 | 0 |
| **Vomiting** | 1(1.7) | 2(2.3) | 0 | 0 |  | 0 | 0 | 1(2.7) | 0 |
| **Diarrhea** | 0 | 2(2.3) | 1(2.7) | 1(6.7) |  | 0 | 1(1.2) | 0 | 0 |
| **Stuffy nose** | 1(1.7) | 2(2.3) | 0 | 0 |  | 1(1.7) | 2(2.3) | 1(2.7) | 0 |
| **Sore throat** | 4(6.9) | 4(4.7) | 2(5.4) | 1(6.7) |  | 4(6.9) | 3(3.5) | 3(8.1) | 2(13.3) |
| **Cough** | 1(1.7) | 0 | 0 | 1(6.7) |  | 3(5.2) | 1(1.2) | 1(2.7) | 1(6.7) |
| **New or worsened muscle pain** | 2(3.4) | 2(2.3) | 2(5.4) | 0 |  | 2(3.4) | 4(4.7) | 1(2.7) | 0 |
| **New or worsened joint pain** | 1(1.7) | 1(1.2) | 0 | 0 |  | 1(1.7) | 1(1.2) | 1(2.7) | 0 |
| **Skin rash** | 3(5.2) | 6(7.0) | 5(13.5) | 1(6.7) |  | 3(5.2) | 5(5.8) | 3(8.1) | 0 |

| **Additional file 1 Table S5.** Anti-SARS-CoV-2 IgG after 4 weeks of the second dose of vaccination in patients with AD and CG. | | | |
| --- | --- | --- | --- |
|  | **AD (n=83)** | **CG (n=308)** | ***P*** |
| S/CO≥2 | 79 (95.2%) | 277 (89.9%) | 0.305 |
| 1<S/CO <2 | 4 (4.8%) | 29 (9.4%) |  |
| S/CO <1 | 0 (0.0%) | 2 (0.7%) |  |
| AD, allergic diseases; CG, control group; S/CO, sample cut-off value | | | |

**Figure legends**

**Additional file 1 Figure S1.** The diagram depicts the enrollment and analysis of participants.


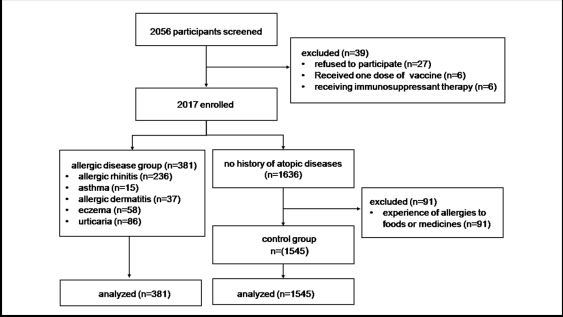

Supplement: Supplementary file 1 — Additional file 1. Table S1. Local reastion grading scale. Table S2. Systemic event grading scale. Table S3. Detailed information of allergic status. Table S4. Local and systemic reactions following vaccination among patients with eczema, uriticaria, aoptic dermatitis, and asthma. Table S5. Anti-SARS-CoV-2 IgG after 4 weeks of the second dose of vaccination in patients with AD and CG. Figure S1. The diagram depicts the enrollment and analysis of participants. [file 12931_2022_2054_MOESM1_ESM.doc]
